# Lack of Medical Criteria for Long-Term Oxygen Therapy Usage According to International Guidance in Outpatients With Chronic Hypoxemia

**DOI:** 10.7759/cureus.19634

**Published:** 2021-11-16

**Authors:** Carlos David Perez-Malagon, Raul Barrera

**Affiliations:** 1 Centro de Ciencias Biomedicas, Universidad Autónoma de Aguascalientes, Aguascalientes, MEX; 2 Clinical Service Rotation, Unidades Médicas de Atención Ambulatoria, Instituto Mexicano del Seguro Social, Aguascalientes, MEX; 3 Autoimmunity, National Institute of Respiratory Diseases, Mexico City, MEX

**Keywords:** survey, respiratory failure, chronic obstructive pulmonary disease, hypoxemia, hypoxemic, long-term oxygen therapy

## Abstract

Background

Benefits of long-term oxygen therapy (LTOT) have been observed in hypoxemic respiratory patients. Reports have shown the lack of observance among healthcare professionals of LTOT. Thus, this study aimed to determine the demographic characteristics and observance of the medical indication of LTOT according to the international guidelines.

Methodology

A cross-sectional study was conducted on patients who attended the Medical Unit in Aguascalientes, Mexico to re-evaluate the need for LTOT. Data are presented as mean ± standard deviation. Statistical analyses were performed using the chi-square test or unpaired t-tests. P-values of <0.05 were considered statistically significant.

Results

From 813 outpatients attended to re-evaluate whether they met the medical criteria to use LTOT, 93 outpatients were excluded, and the remaining 714 outpatients were enrolled. The mean age of the patients was 70.0 ± 15.1 years, with a female gender predominance (59.1 %). The mean PaO_2_ level in room air was 7.9 ± 2.3 kPa. Hemoglobin and hematocrit levels were 14.9 ± 4.1 g/dL and 44.7 ± 8.4%, respectively. The mean levels of PaO_2_ were higher in female patients (8.1 ± 2.5 kPa vs. 7.6 ± 1.9 kPa; p = 0.01). The most common diagnosis was chronic obstructive pulmonary disorder (60.5%). Moreover, the specialty that most indicated the use of LTOT was pulmonology (57.8%); however, 36.8% of patients who used LTOT did not have any criteria according to international guidelines.

Conclusions

Although a significant percentage of patients do not use LTOT correctly, the most important finding is that the medical indication of LTOT by physicians is not always correct, leading to an excessive prescription of oxygen.

## Introduction

Hypoxemia is a state in which oxygen is not available in sufficient quantity in the blood to maintain adequate homeostasis in different organs, leading to a poor prognosis with high mortality; therefore, it must be treated with oxygen therapy [1]. Long-term oxygen therapy (LTOT) refers to the continuous administration of oxygen in patients with chronic hypoxemia to maintain adequate oxygen saturation [2].

Two randomized controlled trials, namely, the Nocturnal Oxygen Therapy Trial (NOTT) and the British Medical Research Council (MRC) trial, conducted in the late 1970s demonstrated that LTOT should be used for patients with chronic obstructive pulmonary disorder (COPD) if their resting awake oxygen tension in arterial blood (PaO_2_) is <7.3 kPa (≤55 mmHg), arterial oxygen saturation (SaO_2_) is ≤88%, PaO_2_ is in the range of 7.3-7.9 kPa (56-59 mmHg), or if SaO_2_ is 89%. Moreover, LTOT should be administered in the presence of signs of cor pulmonale and at a hematocrit level of >55% [3,4]. LTOT has also been extended to COPD patients with resting PaO_2_ of <7.9 kPa (<60 mmHg) and decreased SaO_2_ during exercise or sleep [5]. Both the NOTT and MRC trials reported the following indications for LTOT administration: SaO_2_ of <88% during sleep or a lowering of PaO_2_ to 1.3 kPa (10 mmHg) or more, a fall in SaO_2_ of more than 5% with signs or symptoms suggestive of hypoxemia during exercise, lowering of PaO_2_ to 7.3 kPa (55 mmHg), or lowering of SaO_2_ to 88% [5]. Thus, the goal of LTOT in any of the above indications is to increase the baseline PaO_2_ to at least >8.0 kPa (56-59 mmHg) and/or SaO_2_ to 90% to preserve vital organ functions by ensuring adequate delivery of oxygen [2,3].

The application of LTOT has shown improvement in survival of hypoxemic patients [6] and has been associated with a decrease in vascular pulmonary resistance, pulmonary arterial hypertension, and hematocrit, as well as a reduction in right heart pressure, reduction in minute ventilation, air trapping, and improvement in exercise endurance, thus alleviating post-exercise dyspnea. Nevertheless, clinical trials have not reported benefits from LTOT in the prognosis, rate of hospitalization, or health-related quality of life in COPD patients with mild-to-moderate hypoxemia [4].

The benefits of LTOT for all hypoxemic respiratory patients have been extrapolated from the evidence of its life-saving effects in COPD, and many countries have extended programs for domiciliary oxygen. For example, when nocturnal supplemental oxygen is administered, it improves oxygenation and limits cardiac arrhythmias, prevents high blood pressure, improves the quality of life, and decreases the number of hospital admissions [2-4]. Furthermore, it is generally accepted without much evidence that LTOT in clinical practice is warranted in other forms of chronic respiratory failure such as pulmonary fibrosis, kyphoscoliosis, and cystic fibrosis when arterial blood gas criteria are similar to those established for COPD patients [7].

During our daily medical assistance of patients with chronic respiratory failure, we noticed a lack of observance among medical specialists toward the prescription of LTOT according to international guidelines, a situation which appears to be frequently reported in patients to require LTOT [8]. Thus, we designed a cross-sectional study using survey questionnaires for patients to whom LTOT was previously indicated to determine demographic characteristics and different aspects of the medical indications for LTOT to highlight the lack of observance among medical specialists toward the prescription of LTOT according to international guidelines.

## Materials and methods

We interviewed 813 patients who underwent medical evaluation for eight months at the Outpatient Clinical Medical Unit of Therapy, Aguascalientes, Mexico, a public facility serving the population affiliated to the Mexican Institute of Social Security, to re-evaluate whether they met the medical criteria to continue LTOT.

The criteria of qualification for LTOT were in accordance with policies and procedures of the American Thoracic Society (ATS) [9]. The criteria for LTOT were verified by two pulmonary specialists by checking medical history, physical examination, chest X-ray, electrocardiogram, echocardiography (when necessary), arterial blood gas analysis on room air, hematocrit, and hemoglobin.

The prescription of LTOT was defined as appropriate if at-rest PaO_2_ was ≤7.3 kPa (≤55 mmHg) or when PaO_2_ was 56-59 mmHg or SaO_2_ was ≥89%-90%, combined with radiological or electrocardiographic signs of pulmonary hypertension and/or secondary polycythemia (hematocrit exceeding 55%).

The patient selection criteria for the survey included patients who were prescribed LTOT, those suffering from chronic non-malignant lung diseases, and those referred to the Outpatient Clinical Medical Unit of Therapy for routine follow-up at least twice per year. Additionally, patients with adequate clinical records (arterial blood gas, oxygen administration, and smoking status) were considered eligible.

This study was approved by the Research Ethics Committee of Clinical Medical Unit of Therapy of the IMSS Aguascalientes, Mexico, and all participating patients provided written informed consent.

The results are presented as the arithmetic mean ± standard deviation (SD) or median (interquartile range) and frequencies and percentages for qualitative variables. Comparison between groups was performed using an unpaired t-test for quantitative variables with normal distribution, Mann-Whitney U-rank test for quantitative variables without normal distribution, and the chi-square test was used to compare proportions. Results were considered statistically significant at a p-value of <0.05. The analyses were performed using Stata statistical software package, version 10.0 (StataCorp LP, College Station, TX, USA).

## Results

Over eight months, 813 patients at the Outpatient Clinical Medical Unit of Therapy underwent a routine follow-up to re-evaluate whether they met the medical criteria to use LTOT and continue with oxygen prescription. Of these, 93 outpatients were excluded from the study because they were using supplementary oxygen due to an obstructive apnea syndrome diagnosis, and the remaining 714 outpatients were enrolled in the study. Demographic and clinical characteristics of outpatients attended to in the follow-up clinic are shown in Table 1.

**Table 1:** Baseline and clinical characteristics of the study population. Asterisks indicate differences of statistical significance (*p < 0.05) within the groups determined by the t-test. SD: standard deviation; LTOT: long-term oxygen therapy

	Overall, N = 714	Male, N = 292	Female, N = 422
	Mean ± SD	Mean ± SD	Mean ± SD
Patient age (years)	70.0 ± 15.1	71.4 ± 13.9	68.9 ± 15.9
Time elapsed since diagnosis (years)	7.0 ± 8.8	6.9 ± 9.0	7.1 ± 8.8
Time using LTOT (years)	2.5 ± 3.1	2.3 ± 2.6	2.6 ± 3.3
LTOT prescription (hours/day)	11 ± 6.1	11.2 ± 6.7	10.8 ± 5.6
Oxygen flow (L/minute)	2.5 ± 0.8	2.5 ± 0.7	2.5 ± 0.9
PaO_2_ (kPa)	7.9 ± 2.3	7.6 ± 1.9	8.1 ± 2.5*
PaCO_2_ (kPa)	4.5 ± 0.8	4.6 ± 0.8	4.5 ± 0.8
Hemoglobin (g/dL)	14.9 ± 4.1	15.0 ± 2.6	14.8 ± 4.9
Hematocrit (%)	44.7 ± 8.4	45.6 ± 8.4	44.0 ± 8.4*

The mean age of the study participants was 70.0 ± 15.1 years (range, 19-99 years), with a female gender predominance (422 patients, 59.1%). The average time for LTOT use after therapeutic prescription by a physician was approximately 7.0 ± 8.8 years, while the average time of LTOT use was approximately 2.5 ± 3.1 years. The average daily duration of LTOT in the entire group was 11.1 ± 6.1 hours/day with an oxygen flow of 2.5 ± 0.8 L/minute. The mean PaO_2_ level on room air was 7.9 ± 2.3 kPa (59.6 ± 17.7 mmHg). Hemoglobin and hematocrit levels were 14.9 ± 4.1 g/dL and 44.7 ± 8.4%, respectively.

Female patients were younger than male patients (p = 0.030). Furthermore, no statistically significant differences were noted for the time since diagnosis while using LTOT, daily duration of LTOT, or oxygen flow (Table 1). However, the mean levels of PaO_2_ were higher in female than male patients (8.1 ± 2.5 kPa vs. 7.6 ± 1.9 kPa; p = 0.01), whereas hematocrit, although almost normal, was slightly lower (44.0 ± 8.4 % vs. 45.6 ± 8.4%; p = 0.0125).

In this survey, we found that the most common diagnosis was COPD (60.5%), followed by other diagnoses, each with a prevalence of less than 10% (Table 2).

**Table 2:** The total number of patients according to the respiratory diagnosis. COPD: chronic obstructive pulmonary disease; ILD: diffuse interstitial lung disease; OSA: obstructive sleep apnea; SD: standard deviation

	Overall, N (%)	Male, N (%)	Female, N (%)
COPD	432 (60.5)	188 (64.4)	244 (57.8)
ILD	60 (8.4)	25 (8.6)	35 (8.3)
OSA	36 (5.0)	15 (5.1)	21 (5.0)
Heart failure	29 (4.1)	12 (4.1)	17 (4.0)
Renal insufficiency	15 (2.1)	4 (1.4)	11 (2.6)
Lung cancer	15 (2.1)	8 (2.7)	7 (1.7)
ILD + other	5 (0.7)	1 (0.3)	4 (0.9)
Bronchiectasis	1 (0.1)	0 (0.0)	1 (0.2)
Others	121 (16.9)	39 (13.4)	82 (19.4)

For these patients, the medical specialty that most indicated LTOT administration was pulmonologists (57.8%), followed by internists (20.3%) and cardiologists (9.2%), although other specialties also indicated LTOT (Table 3).

**Table 3:** Specialists who prescribed LTOT to patients according to international criteria. Asterisks indicate the percentage regarding the number of patients with criteria positive or negative for LTOT. LTOT: long-term oxygen therapy

	Prescription of LTOT, n (%)	Criteria of LTOT	
		Yes, n (%*)	Not, n (%*)
Pulmonologist	413 (57.8)	267 (59.2)	146 (55.5)
Internist	145 (20.3)	84 (18.6)	61 (23.2)
Cardiologist	66 (9.2)	41 (9.1)	25 (9.5)
Nephrologist	8 (1.1)	4 (0.9)	4 (1.5)
Geriatrics	7 (1.0)	5 (1.1)	2 (0.8)
Neurologist	2 (0.3)	1 (0.2)	1 (0.4)
Ear nose throat	1 (0.1)	1 (0.2)	0 (0.0)
Rheumatologist	1 (0.1)	1 (0.2)	0 (0.0)
Surgeon	1 (0.1)	1 (0.2)	0 (0.0)
Oncologist	1 (0.1)	0 (0.0)	1 (0.4)
Unknown	69 (9.7)	46 (10.2)	23 (8.7)

When we compared study variables between those who met the criteria for LTOT with those who did not, we observed that there was no difference in age, the time elapsed with chronic hypoxemia, the time using LTOT, and oxygen flow (Table 4).

**Table 4:** Comparison between patients with chronic hypoxemia who qualified or did not qualify for the criteria recommended by LTOT guidelines. Asterisks indicate the differences in statistical significance (*p < 0.05) within the groups determined by the t-test. LTOT: long-term oxygen therapy; SD: standard deviation

	Criteria for prescription of LTOT	
	Positive (N = 451; 63.2%)	Negative (N = 263; 36.8%)
	Mean ± SD	Mean ± SD
Age (years)	70.1 ± 15	69.7 ± 15.4
Time elapsed since diagnosis (years)	7.4 ± 8.6	6.4 ± 9.2
Time using LTOT (years)	2.6 ± 3.0	2.1 ± 3.1
LTOT prescription (hours/day)	11.3 ± 5.2	10.4 ± 7.3
Oxygen flow (L/minute)	2.5 ± 0.8	2.5 ± 0.8
PaO_2_ (kPa)	7.0 ± 1.67	9.5 ± 2.67*
PaCO_2_ (kPa)	4.7 ± 0.89	4.2 ± 0.77*
Hemoglobin (g/dL)	15.6 ± 4.8	13.6 ± 2.2*
Hematocrit (%)	46.7 ± 8.9	41.1 ± 6.2*

## Discussion

The benefits of LTOT in patients with chronic hypoxemia have been demonstrated in numerous studies, with improved survival being the most crucial [10]. Other benefits of LTOT include improved quality of life, improved exercise capacity, improved intellectual function and mental status, reduced secondary polycythemia, and stabilized pulmonary artery pressure [11].

Our surveys results showed that the mean age of outpatients using LTOT was 70.0 ± 15.1 years (range, 19-99 years), with patients largely suffering from chronic hypoxemia. This wide range is due to the inclusion of young patients who require LTOT for different diseases such as bronchiectasis and congenital cardiovascular, renal, and other diseases. The mean age was similar to previous studies. For example, Nasilowski et al. [12] in Poland reported a study of 30 patients (77% with COPD) with a mean age of 67 ± 9 years, whereas Neri et al. [13], in a multicenter observational study conducted in Italy (1,504 patients, 74% COPD), found a mean age of 71.6 ± 10 years. Similarly, Mesquita et al. [14], in a study made in Brazil with 39 patients with COPD and exertional dyspnea, reported a mean age of 69.0 years (62.0-77 years). In a study done in Denmark including 182 patients with LTOT (84.1% with COPD), Ringbaek et al. [15] in a report of 246 COPD patients found that those who received continuous oxygen therapy were younger than those who received non-continuous oxygen therapy (mean age: 68.4 ± 8.7 vs 71.6 ± 7.4 years, respectively). Subsequently, in 2014, the same group evaluated the changes among 14,965 COPD patients treated with LTOT from 2001 to 2010 and reported that the mean age increased from 73.4 ± 9.0 years to 74.8 ± 9.7 years [16].

Our study showed that 60.5% of outpatients with an LTOT prescription were diagnosed with COPD, which is a slightly lower percentage than that reported in other studies. For example, in Denmark, Ringbaek et al. [17] found that 84% of all surveyed patients who used LTOT had COPD, and similar percentages were reported by Nasilowski et al. [14] in Poland (77%), Katsenos et al. [18] in Greece (74.7%), Chaney et al. [19] in the United States (75%), and Neri et al. [13] in Italy (74%), whereas Coleta et al. [20], in Brazil, reported that 67.8-81.6% of all patients treated with LTOT had COPD. Interestingly, Rico-Mendez et al. [21] in a study done in Mexico with patients receiving home-based LTOT reported that only 44% had a diagnosis of COPD.

Female patients predominated in our study (59.1%) and were younger than male patients (females: 68.9 ± 15.9 years vs. male: 71.4 ± 13.9 years; p = 0.030). In a seven-year prospective cohort study of 435 outpatients with COPD conducted in Brazil, Machado et al. [22] observed that females were significantly younger than males (62.9 ± 6.5 vs. 69.3 ± 7.2 years, respectively), highlighting that females with severe COPD using LTOT for more than three years were at a higher risk of death. Likewise, Ringbaek et al. [15] in 2002 reported female predominance depending on their use of oxygen (continuous 56.8% vs. 48.8 non-continuous oxygen usage), although they did not report differences in age in relation to gender.

A significant number of our patients (69.2%) received the prescription for oxygen use for an average of 11 hours/day. Much of the initial research for LTOT addressed the precision of the daily duration of LTOT after therapeutic prescription. In particular, the prescription of oxygen administration for at least 15 hours/day is recommended according to current guidelines for patients with chronic hypoxemia (PaO_2_ ≤ 55 mmHg) because this treatment was found to reduce overall mortality compared to no LTOT (33% vs. 55%; p < 0.05) [23].

However, despite the generally recommended daily duration of oxygen usage to achieve its goals, there is a large range of mean time between different surveys. For example, Atiş et al. [24] found that Turkish patients used LTOT for 9.7 ± 6.09 hours/day, which is equivalent to the study reported by Katsenos et al. [18] (9 ± 6.8 hours/day), whereas Mesquita et al. [14] reported that the mean daily hours of using LTOT was as low as 8.0 ± 1.2 hours/day and Nasilowsky et al. [12] reported that all patients used LTOT for an average of 12.5 ± 4.6 hours/day. However, they reported that only 37% of patients followed the prescription properly (mean oxygen use: 17.4 ± 2.6 hours/day), and in patients who did not adhere to prescription, the mean duration was 9.6 ± 2.7 hours/day. In other studies, Neri et al. [13] found that 79% of their patients used LTOT for more than 15 hours/day, whereas Gauthier et al. [25] in Canada reported that 115 patients were exposed to LTOT for an average of 17.8 hours/day, reaching the suggested daily hours by international guidelines; nevertheless, 40% of patients did not use LTOT according to their medical prescription. Ringbaek et al. [17] found that 65% of patients used LTOT for more than 15 hours/day, and 29% of the study participants used oxygen for fewer than 15 hours/day. In a subsequent study, the same group reported that adherent patients used LTOT for 18.5 ± 3.4 hours/day, whereas in nonadherent patients the duration was 8.2 ± 4.8 hours/day [15]. These large differences might be due to the observance of physicians and sociocultural aspects of patients.

The most common dose of LTOT used by our outpatients was 2.5 L/minute, but doses higher than 3 L/minute were also used in 15% of cases, compared to the 10% reported by Neri et al. [13]. Although the NOTT study recommended that COPD hypoxemic patients can be prescribed 1-2 L/minute of LTOT and less than 10% of cases might require 3 L/minute or more to achieve adequate oxygenation, Wijkstra et al. [7] stated that there are substantial differences among countries as to how clinicians should choose the flow rates patients should receive at rest, during sleep, and during exercise. Thus, Ringbaek et al. [17] found that most patients were prescribed a flow of 1 L/minute, and only 7% were prescribed more than 1.5 L/minute. Another study found that adherent and nonadherent LTOT patients used 1.3 ± 0.6 to 1.3 ± 0.7 L/minute, a low dose than the recommended guidelines [17], whereas Cherniack et al. [26] and Bigelow et al. [27] proposed that oxygen flows for LTOT can be set at 2 L/minute by nasal cannulae. Unfortunately, we did not investigate this in our study.

We found that the main medical specialists who recommended LTOT to patients were pulmonologists in 57.8%, internists in 20.3%, and other specialists in 12.0% of the patients. However, the proportion of patients who were appropriately prescribed according to LTOT guidelines was 63.1%. Pulmonologists often presented better adherence to the guidelines (59.2%) compared to other specialists. Similar data were reported by Verduri et al. [28] who reported that LTOT was prescribed by a pulmonologist in 64.5%, by an internist in 29.3%, and by other specialists in 6.2% of the patients. While this study appeared to show that the specialty of the prescriber had no impact on the appropriateness of the prescription, we considered that pulmonologists have a greater opportunity to give patients more detailed explanations to improve their understanding and compliance to treatment, making educating physicians, patients, and relatives regarding the use of LTOT crucial.

While there is a broad agreement based on resting PaO_2_ as to who should receive LTOT, we observed that a significant proportion of patients received LTOT prescription for hypoxemia without meeting the criteria of LTOT guidelines. A significant number of patients (36.8%) had mean PaO_2_ higher than 8 kPa (9.5± 2.67 kPa) at the last visit. Our study, that evaluated the medical criteria for LTOT use previously established by LTOT guidelines showed that 36.8% of patients did not meet the proposed criteria for medical prescription of LTOT. These findings are a cause of concern because they do not correspond to evidence-based recommendations for good clinical practice.

The lack of observance of LTOT criteria appears to be a frequent practice in several studies concerning LTOT. Thus, several series in different countries have reported a significant percentage of patients with LTOT who are incorrectly prescribed LTOT. Studies documenting patients with erroneous prescriptions of LTOT have been reported by Morrison et al. [29] in Scotland (86%), Munilla et al. [30] in Spain (28.5-46%), Granados et al. [31] in Spain (42%), and Guyatt et al. [32] in Canada (40.5%). A French study showed that 18% of 7,700 patients who were on LTOT had a PaO_2_ of >8.0 kPa [33]. In later studies, Chaney et al. [19] observed that 50.5% of patients with LTOT did not require it in the first evaluation, and in the second evaluation, 58% of them no longer met the criteria for LTOT. Magnet et al. [34] in Germany found that using a limit value for LTOT at 55 mmHg or 60 mmHg, 21% or 30% of patients, respectively, were overprescribed with LTOT. Additionally, in the study by Rico-Méndez et al. [21], 41.9% of the population prescribed LTOT was lacking arterial blood gases (PaO_2_ or SaO_2_), so the prescription of LTOT in patients without COPD was inappropriate. These studies demonstrate an abuse of LTOT prescription worldwide; hence, LTOT should only be administered for strict indications, in accordance with the guidelines, and only in a form suitable for individual patients.

It is important to consider that patients with prescription of LTOT often show poor adherence, ranging from 45% to 70% [12,17,24]. The lack of precise instructions for LTOT prescription limits patient adherence. Adherence is likely to be a central determinant for the effectiveness of LTOT, and more research is needed to evaluate and improve adherence to LTOT. Walshaw et al. [35] concluded that effective prescription and compliance were associated with a respiratory physician more often than a family doctor. Furthermore, regarding erroneous oxygen prescriptions, oxygen costs (nearly 50 dls per 10,000 L in Mexico) are increasing due to the inappropriate use of oxygen [36].

Our survey results, like others, showed that LTOT is frequently prescribed without following the LTOT guidelines, regardless of the medical specialty. Patients need to be able to meet the criteria for LTOT, and they must be clinically stable and should be receiving optimal pharmacological treatment prior to commencement. LTOT is frequently prescribed in an unstable clinical situation or after a single assessment, as reported by several authors [37]. Howard et al. [38] reported that physicians varied widely in their prescribing habits. Approximately 36% of LTOT patients were prescribed less than 15 hours/day, thus reducing the optimal dosage. Another study found that 55% of patients had not received thorough written instructions regarding the use of LTOT by their physician, and 63% were not aware of the importance of LTOT in the therapeutic management of their disease [18]. However, two randomized clinical trials have shown that even if LTOT is appropriately prescribed, it is not as effective as expected.

The Long-Term Oxygen Treatment Trial, a large randomized clinical trial of long-term supplemental oxygen versus no long-term supplemental oxygen in patients with COPD, demonstrated no benefit with respect to survival or first hospitalization during one to six years of follow-up [39]. Lacasse et al., in a double-blind, placebo-controlled, randomized trial, showed no difference in patients with stable COPD regarding survival, rates of exacerbation, hospitalization, and quality of life with nocturnal oxygen therapy in three to four years of follow-up [40].

Limitations

The limited sample of interviewed patients makes the results difficult to generalize to all local, regional, or national prescriptions. Another limitation was the lack of information concerning the measurement of blood saturation at night and during exercise. Other limitations include adherence to the time of use, used/prescribed oxygen flow, and the fact that during the follow-up the clinical condition of the patient could have changed, which may explain, at least in part, the lack of adherence to the LTOT criteria. Nonetheless, the study provides important preliminary information to design and perform more accurate and larger studies to improve the appropriate observance of LTOT guidance in clinical practice.

## Conclusions

According to our results, we can conclude that there is a significant percentage of patients who do not use LTOT correctly. Moreover, we found that the medical indication of LTOT by physicians is not always correct as they do not base their decision regarding prescribing oxygen on international criteria, which occurs in different regions of the world. The most important finding was that when LTOT is prescribed without any medical criteria, there is a clear non-necessity of LTOT in several patients. This study highlights the importance of establishing healthcare plans that encourage the proper management of LTOT.
